# Respiratory Complications Are the Main Predictors of 1-Year Mortality in Patients with Hip Fractures: The Results from the Alzira Retrospective Cohort Study

**DOI:** 10.3390/geriatrics9020047

**Published:** 2024-04-09

**Authors:** Elisa García-Tercero, Ángel Belenguer-Varea, Daniela Villalon-Ruibio, Jesús López Gómez, Rodrigo Trigo-Suarez, Cristina Cunha-Pérez, Miguel Germán Borda, Francisco Jose Tarazona-Santabalbina

**Affiliations:** 1 Geriatric Medicine Department, Hospital Universitario de la Ribera, Carretera de Corbera km. 1, 46600 Alzira, Spain; abelenguervarea@gmail.com (Á.B.-V.); dandrea.villalon@gmail.com (D.V.-R.); jblg93@gmail.com (J.L.G.); fjtarazonas@gmail.com (F.J.T.-S.); 2 School of Doctorate, Universidad Católica de Valencia San Vicente Mártir, 46001 Valencia, Spain; crizzia@gmail.com; 3 Centre for Age-Related Medicine (SESAM), Stavanger University Hospital, 4068 Stavanger, Norway; mmborda@gmail.com; 4 Semillero de Neurociencias y Envejecimiento, Ageing Institute, Medical School, Pontificia Universidad Javeriana, Bogotá 110231, Colombia; 5 Division of Clinical Geriatrics, Center for Alzheimer Research, Department of Neurobiology, Care Sciences and Society, Karolinska Institutet, 14186 Stockholm, Sweden; 6 Centro de Investigación Biomédica en Red Fragilidad y Envejecimiento Saludable (CIBERFES), 28029 Madrid, Spain; 7 Medical School, Universidad Católica de Valencia Sant Vicent Màrtir, 46001 Valencia, Spain

**Keywords:** hip fracture, older adults, 1-year mortality, risk factors, orthogeriatric care, fragility fractures

## Abstract

**Introduction**: Hip fractures pose a significant challenge for older individuals given their high incidence and one-year mortality rate. The objective of this study was to identify the primary predictors of one-year mortality in older adults hospitalized for hip fractures. **Methods:** We conducted a retrospective cohort study involving adults aged 70 years or older who were admitted to the hospital for fragility hip fractures between 1 January 2014 and 31 December 2021. A total of 3229 patients were recruited, with 846 (26.2%) experiencing one-year mortality. **Results:** Respiratory complications (HR 2.42, 95%CI 1.42–4.14; *p* = 0.001) were the most significant predictors of one-year mortality, followed by hospital readmission (HR 1.96, 95%CI 1.66–2.32; *p* < 0.001), the male sex (HR 1.88, 95%CI 1.46–2.32; *p* < 0.001), cardiac complications (HR 1.88, 95%CI 1.46–2.32; *p* < 0.001), and a diagnosis of dementia at admission (HR 1.37, 95%CI 1.13–1.66; *p* = 0.001). The Charlson Index and the American Society of Anesthesiologists physical status classification system also significantly increased the mortality risk. Conversely, higher hemoglobin levels at admission and elevated albumin at discharge significantly reduced the mortality risk. **Conclusions:** The one-year mortality rate is substantial in older adults with hip fractures who are admitted to an orthogeriatric unit. The appropriate management of anemia, nutritional disorders, and comorbidity at admission and during the follow-up could potentially mitigate long-term mortality after hip fractures.

## 1. Introduction

Hip fracture stands as a significant contemporary health challenge, representing the most severe osteoporotic complication for both individuals and healthcare systems. In Spain, its incidence is approximately 104 cases per 100,000 inhabitants, and this number is anticipated to rise in the forthcoming years due to the aging population, thereby escalating the prevalence of osteoporosis and the risk of fracture among individuals over 80 years [1]. This age cohort is particularly susceptible to postoperative medical and surgical complications, including pneumonia, delirium, heart failure, and urinary tract infections [2,3].

The nature of the affected demographic, coupled with the inherent attributes of the clinical condition and the consequential complications stemming from surgical interventions and subsequent hospital stays, significantly elevates the likelihood of disability and mortality in the short, medium, and long term for individuals afflicted with hip fractures. Studies examining the mortality risk after a hip fracture suggest a mortality rate of approximately 4.5% during hospitalization [4] and 20–30% one year after hospital discharge [5,6].

The concerning mortality rate, paired with the expected increase in osteoporotic fractures in the future, emphasizes the necessity of identifying potential risk factors for mortality in patients with hip fractures. In recent years, numerous factors have been elucidated that significantly impact morbidity and mortality in individuals with hip fractures. These encompass an advanced age, the male sex, prior institutionalization, functional decline, delayed surgery, cognitive status, delirium, and increased comorbidity [3,6,7,8,9,10]. A meta-analysis conducted by Chang et al. revealed that surgical delays exceeding two days, along with the presence of COPD, diabetes, cardiovascular disease, and prior institutionalization, were significant contributors to an increased risk of mortality [11]. Nevertheless, the specific hierarchy of risk among these factors remains unclear, and it is yet to be determined whether, once identified, they could potentially be preventable or treatable.

This study aims to identify the risk factors for long-term mortality in older adults with hip fractures, encompassing anesthetic risk scales and comorbidity indices. Additionally, this study aims to determine which factors hold the highest predictive power, thus enabling early intervention strategies.

## 2. Methods

This study is designed as an observational, analytical investigation within a retrospective cohort of older adults who were admitted to an orthogeriatric unit in the hospital due to hip fracture.

### 2.1. Scope and Population

The Hospital Universitario de la Ribera (HULR) serves a population of 305,085 inhabitants in La Ribera (Alzira, Valencian Community, Spain), with 19% being over 65 years old. In the surgical domain, geriatricians attend to all patients aged 70 years or older and those who were admitted for hip fractures. In other diagnostic areas, such as traumatology, general surgery, vascular surgery, neurosurgery, and urology, a risk screening is conducted based on the pre-anesthetic assessment of elective procedures or the consideration of comorbidities, functional status, and the patient’s clinical condition upon admission to the Emergency Department (ED) [12].

### 2.2. Eligibility Criteria

Inclusion criteria encompassed patients aged 70 or older who were admitted to the hospital due to a hip fracture following a low-impact fall. Exclusion criteria involved a diagnosis of polytrauma upon admission, admission with a diagnosis of pathological fracture, and an estimated life expectancy of less than six months from any cause.

### 2.3. Sample Size

All patients aged 70 or older who were admitted to the Hospital Universitario de La Ribera (Alzira, Valencia, Spain) for a hip fracture between 1 January 2014 and 31 December 2021 were consecutively included. A sample of 3229 patients was obtained. The power of this study was calculated by estimating a proportion of males of 35% in the group of deceased individuals and 28% in the group of survivors, with an alpha error of 5%, which resulted in a power calculation of 96.2%.

### 2.4. Intervention

Following the diagnosis of a hip fracture in the hospital, each patient was allocated to an orthogeriatric team comprising a traumatologist, a geriatrician, and a team of nurses for the entire duration of the hospital stay. The geriatrician and orthopedic surgeon conducted an assessment within the initial 24 h, followed by daily assessments until discharge. After surgery, the rehabilitation service team evaluated the patient and initiated rehabilitation therapy within 24 h of the surgical procedure [12].

### 2.5. Study Variables

Data were sourced from the electronic medical record using the SIAS© software 20.7.7. Information was extracted from the medical records and hospital discharge summaries of patients diagnosed with hip fractures during the study period.

Follow-up data were gathered from outpatient records, emergency care episodes, and post-discharge hospital admissions. Subjects lacking follow-up data within 12 months after discharge were contacted by telephone. Survival data were acquired from medical records, where patient deaths within the care area are updated daily. Two co-investigators, blind to the design and objectives of this study, were responsible for collecting these data.

Sociodemographic variables, such as age and sex, surgical delay, hospital stay, comorbidities measured according to the Charlson Comorbidity Index (CCI) [13], the age-adjusted Charlson Comorbidity Index, the Cumulative Illness Rating Scale-Geriatric (CIRS-G) [14], the American Society of Anesthesiologist (ASA) classification, the nutritional control scale (CONUT) [15], the presence of geriatric syndromes such as delirium, the transfusion of red blood cell units, and medical and surgical complications during admission, along with 12-month mortality, were evaluated.

### 2.6. Statistical Analysis

Variable values were entered into an Excel spreadsheet, version 2007, and a statistical analysis was performed using IBM SPSS Statistics for Windows, Version 23.0 (Armonk, NY, USA: IBM Corp.). Quantitative variables were expressed as the mean or median of the central position, and the measure of dispersion was expressed as the standard deviation or interquartile range depending on whether or not the variables met the criteria for normal distribution. Qualitative variables were expressed as total case numbers and percentages. Bivariate analysis was performed using Student’s *t*-test (Fisher method) for quantitative variables with normal distribution, and Pearson’s chi-squared test and Mantel–Haenszel linear trend were used for proportions for qualitative variables. Non-parametric techniques were used for quantitative variables that did not meet the criteria for normal distribution.

Variables associated with one-year mortality were analyzed using a Cox proportional hazards model, including deaths observed during the 365 days after admission for hip fracture. First, the entire model was considered, including all variables that were significantly associated with one-year mortality in the bivariate analysis. In the second step, any variable that did not produce a significant change (defined as the absence of an adjusted effect more significant than 10%) or that did not improve the standard error of the model estimate without that variable was removed from the model. In the case of two or more subsets of variables with similar levels of fit, the agreement between the investigators was required to decide which subset to select based on clinical utility criteria. The level of statistical significance was set at *p* < 0.05.

### 2.7. Ethical Considerations

This study complies with the principles of the Declaration of Helsinki and the provisions of the Organic Law on the Protection of Personal Data and the Guarantee of Digital Rights for the Protection of Personal Data (Organic Law 3/2018). It was also approved by the Ethics and Clinical Research Committee of the HULR (reference code HULR23122022).

## 3. Results

### 3.1. Descriptive and Bivariate Analyses

Out of the 3229 patients enrolled, 846 (26.2%) died within one year. The deceased group predominantly comprised males and exhibited a statistically significantly higher age, increased rates of major and total complications, more readmissions, elevated re-intervention rates, increased instances of surgery after the initial 72 h of admission, a higher percentage of patients with an ASA classification ≥2, poorer nutritional status assessed by CONUT at the baseline and the end of hospitalization, and a greater prevalence of comorbidities, such as ischemic heart disease, COPD, heart failure, stroke, dementia, chronic renal failure, and diabetes at admission.

Furthermore, the deceased patients demonstrated higher scores on the Charlson and age-adjusted Charlson comorbidity indices and G-CRIS. They also exhibited higher incidence rates of delirium, cardiac complications, anemia, a higher percentage requiring red blood cell transfusion, digestive complications, urinary tract infections, respiratory complications, and surgical site infections. These conditions were associated with more extended hospital stays and more patients requiring admission to intensive care. Table 1 and Table 2 provide a comprehensive overview of the results for the main variables studied.

Given the elevated mortality risk observed in men, a bivariate analysis was conducted with comorbidity indices and the surgical risk scale. The results indicated that, compared to women, men had significantly higher scores in the Charlson index (3.2 [2.7] vs. 2.3 [2.2]; *p* < 0.001), age-adjusted Charlson index (7.1 [2.8] vs. 6.1 [2.4]; *p* < 0.001), and CIRS-G (12.0 [2.8] vs. 11.2 [2.4]; *p* < 0.001), and there was a higher proportion of male patients with an ASA score of ≥3 (50 [6.8%] vs. 88 [4.4%]; *p* = 0.013). A similar analysis was replicated by grouping the patients by mean age, revealing that those over 85 years had significantly higher Charlson index scores (2.6 [2.6] vs. 2 [2.6]; *p* = 0.013), age-adjusted Charlson index scores (6.7 [2.3] vs. 6.0 [2.8]; *p* < 0.001), and CIRS-G (11.9 [3.8] vs. 10.6 [4.1]; *p* < 0.001), with a larger proportion of patients with an ASA score of ≥3 (77 [5.8%] vs. 52 [4.1%]; *p* = 0.046).

### 3.2. Multivariate Cox Regression

The multivariate cox regression analysis of the variables present upon admission showed that age, the male sex, a history of dementia, an ASA classification ≥3, and the Charlson Comorbidity Index score increased the risk of mortality. In contrast, higher albumin and hemoglobin levels upon admission decreased the chance of mortality (Table 3).

The Cox regression, repeated with the variables that were present at discharge, indicated that the number of complications, hospital readmissions, CONUT score at discharge, and cardiac complications increased the mortality risk (Table 4).

A new multivariate analysis was performed using the key variables identified upon admission and hospital discharge to discern those that were the most strongly associated with one-year mortality, and the results were consistent (Table 5).

## 4. Discussion

Our findings revealed that the most significant predictors of one-year mortality were respiratory complications, followed by hospital readmissions, cardiac complications, male gender, the presence of dementia at admission, and a higher surgical risk estimated by the ASA, Charlson Comorbidity Index score, and age. Higher hemoglobin values at admission and higher albumin values at discharge were identified as analytical parameters that mitigated mortality in our study. 

Notably, the one-year mortality rate was 26.2%. this aligns with rates reported in comparable studies [16,17], including one conducted by our group a decade earlier [12]. With more than one in four patients dying during the year following admission for hip fracture, there is a pressing need for closer post-hospital discharge follow-up, particularly for patients who are at the highest risk.

Identifying patients who are at the most significant risk is crucial for establishing targeted and coordinated care plans across different healthcare levels to mitigate the high mortality observed twelve months after the fracture. Key variables in this identification process include certain complications and hospital readmissions.

This study underscores the significance of respiratory complications during admission, revealing a twofold increase in the risk of death within one year, closely trailed by hospital readmission and cardiac complications. Notably, respiratory infections and heart failure have previously been identified as risk factors for mortality at six months post-hip fracture [18]. Likewise, readmissions have been linked to an elevated mortality risk at one year [19]. The identification of these events underscores the importance of implementing special surveillance for patients presenting with such complications, emphasizing the need for targeted monitoring and intervention strategies to improve outcomes and reduce mortality in this vulnerable population.

Similarly, the male sex showed a higher risk of mortality, which was associated with a higher score in the comorbidity indices evaluated in this study. The incidence of fractures is lower in men than in women. Still, the high level of comorbidity in men significantly increases the risk of mortality [12,20,21], and this calls for greater vigilance in this group of patients.

These comorbidities include a history of dementia [3,7,12,17,18], which significantly increases the risk of death at one year because dementia increases the risk of potentially fatal complications, such as delirium, postoperative infections, and respiratory complications [7].

Surgical risk scales and comorbidity indices are indirect indicators of potential patient risk [22]. In our study, only an ASA classification ≥ 3 and the Charlson Comorbidity Index score maintained statistical significance in the multivariate analysis. Both ASA and the male gender have previously been associated with an increased risk of complications [20]. Similarly, the CCI has shown a strong association with 1-year mortality risk [23,24,25], and a recent paper also found a strong association between 1-year mortality and the male gender, ASA score, and age [17]. Regarding the CIRS-G, a previous study [25] found a relationship between this comorbidity index and mortality, but in patients with longer surgical delays and older ages, which may have favored this association. Age has been associated with more significant mortality one year after the fracture [3,21,26,27]. However, we are not talking about chronological age per se, as the greater prevalence of comorbidities in these older patients, who present significantly more comorbidities estimated in the indices studied, increases the risk of mortality.

Finally, two factors reduce the mortality risk at one year: serum albumin and hemoglobin levels. Serum albumin levels have been linked to the health statuses of older adults and, in the case of lower serum levels, to a more pro-inflammatory state, which increases the risk of postoperative complications, mainly infectious ones [28], and increases the risk of mortality one year after the fracture [29,30]. Hemoglobin levels have also been associated with survival in these patients [17], highlighting the need for the close monitoring of hemoglobin levels upon admission and upon the initiation of combined therapies with intravenous iron, the transfusion of red blood cell concentrates, and perhaps, in the future, erythropoietin, if the evidence supports it, to at least maintain preoperative plasma hemoglobin levels above 10 g/dL to reduce the risk of mortality [31]. Other studies have shown how the need for the transfusion of packed red blood cells increases the risk of mortality [22]. These findings underscore the need for multifactorial peri- and post operative interventions encompassing nutrition supplementation, adequate analgesia, attention to, and treatment of infection, as well as hydration and hypovolemia.

The main limitation of this study is the retrospective design of the analysis of the cases, which may introduce biases and limit the establishment of causal relationships. Loss of follow-up during admission represents less than 2% of cases and is due to the care of patients from other regions or countries who presented with fractures in our area without a subsequent follow-up in our center. This study was conducted in a single hospital, which might impact the generalizability of the findings to a broader population.

However, this study boasts several strengths. Firstly, it features a large sample size, encompassing a substantial number of patients with hip fractures, thereby enhancing the statistical power and reliability of the findings. Additionally, it conducts a comprehensive analysis by exploring various factors, including comorbidity indices, surgical risk scales, and biochemical parameters, providing a holistic view of the participants. This study also incorporates a longitudinal follow-up, enabling the examination of one-year outcomes. Most importantly, this study’s findings contribute valuable insights into the predictors of one-year mortality in older adults with hip fractures, informing clinical decision making and potential interventions. Notably, this study sheds light on a frequent and preventable problem—respiratory complications. These considerations underscore this study’s robustness while acknowledging the potential limitations inherent in observational research.

## 5. Conclusions

One-year mortality was high in older patients with hip fractures who were admitted to a geriatric unit. The leading causes of mortality at one year were respiratory complications, readmission, cardiac complications, the male sex, dementia, age, and lower albumin and hemoglobin levels. The appropriate management of anemia, nutritional disorders, and comorbidity upon admission and during the follow-up could reduce long-term mortality after a hip fracture.

## Figures and Tables

**Table 1:** Bivariate analysis of variables present at hospital admission.

Variable	Survivors n = 2380	Deceased n = 846	*p*-Value
Age (years), m (SD)	83.5 (6.1)	86.7 (6.2)	<0.001
Male sex, n (%)	529 (22.2%)	318 (37.6%)	<0.001
**Biochemical**			
Hemoglobin upon admission (g/dL), m (SD)	12.5 (1.6)	11.9 (1.9)	<0.001
Albumin upon admission (g/dL), m (SD)	3.9 (0.4)	3.8 (0.5)	<0.001
**Clinical**			
Charlson Comorbidity Index, m (SD)	2.2 (2.1)	3.5 (2.7)	<0.001
Initial CONUT, m (SD)	2.9 (1.9)	3.7 (2.1)	<0.001
CIRS-G, m (SD)	10.2 (3.9)	12.0 (4.1)	<0.001
ICC with age, m (SD)	5.8 (2.3)	7.3 (2.8)	<0.001
Glomerular filtration rate upon admission (mL/min), m (SD)	66.0 (24.3)	59.9 (26.5)	<0.001
Initial CONUT with moderate or severe nutritional risk, n (%)	299 (14.5%)	218 (29.4%)	<0.001
Previous ischemic heart disease, n (%)	173 (7.3%)	81 (9.6%)	0.037
Previous heart failure, n (%)	127 (5.3%)	73 (8.6%)	<0.001
Previous COPD, n (%)	323 (13.6%)	168 (19.9%)	<0.001
Previous chronic renal failure, n (%)	217 (9.1%)	133 (15.7%)	<0.001
Previous diabetes, n (%)	651 (27.4%)	264 (31.2%)	0.037

Legend m = mean, SD = standard deviation, n = number, % = percentage, CONUT = controlling nutritional status, CIRS-G = Cumulative Illness Rating Scale-Geriatrics, CCI = Charlson Comorbidity Index, COPD = chronic obstructive pulmonary disease.

**Table 2:** Bivariate analysis of variables analyzed during hospital stay and at hospital discharge.

Variable	Survivors n = 2380	Deceased n = 846	*p*-Value
**Clinical**			
Length of stay, m (SD)	7.8 (3.0)	8.9 (5.9)	<0.001
Number of complications, m (SD)	1.6 (1.8)	0.9 (1.2)	<0.001
Number of major complications, m (SD)	1.4 (1.6)	0.8 (1.1)	<0.001
Days until re-entry, m (SD)	80.1 (83.8)	55.4 (63.6)	<0.001
Surgical delay, m (SD)	44.0 (28.3)	48.9 (38.3)	<0.001
Number of concentrates transfused, m (SD)	1.4 (1.7)	2.0 (2.2)	<0.001
Complication during admission, n (%)	1085 (45.6%)	532 (62.9%)	<0.001
Major complication during admission, n (%)	1047 (44%)	512 (60.5%)	<0.001
Hospital readmissions, n (%)	357 (15%)	298 (35.2%)	<0.001
Reintervention, n (%)	29 (1.2%)	23 (2.7%)	0.006
Intervention first 36 h, n (%)	1528 (64.2%)	511 (60.4%)	0.051
Intervention first 72 h, n (%)	2026 (85.1%)	687 (81.2%)	0.009
ASA score >2, n (%)	69 (3.4%)	69 (9.4%)	<0.001
Patients transfused, n (%)	1335 (56.1%)	573 (67.7%)	<0.001
Final CONUT with moderate or severe nutritional risk, n (%)	362 (17.6%)	273 (36.8%)	<0.001
Delirium (%)	225 (9.5%)	128 (15.1%)	<0.001
Cardiac complication during admission, n (%)	95 (4%)	110 (13%)	<0.001
Final CONUT, m (SD)	2.7 (2.1)	4.0 (2.5)	<0.001
Respiratory complications	61 (2.6%)	97 (11.5%)	<0.001
**Biochemical**			
Hemoglobin at discharge (g/dL), m (SD)	10.3 (1.2)	10.4 (1.6)	0.796
Albumin at discharge (g/dL), m (SD)	3.8 (0.5)	3.6 (0.5)	<0.001
Glomerular filtration rate at discharge (mL/min), m (SD)	82.3 (36.4)	72.8 (39.1)	<0.001

Legend: m = mean, SD = standard deviation, n = number, % = percentage, CONUT = controlling nutritional status, ASA = American Society of Anesthesiology.

**Table 3:** Multivariate Cox regression analysis of variables present upon admission.

Variable	Hazard Ratio	95%CI	*p*-Value
Sex	1.72	1.46–2.03	<0.001
ASA score ≥3	1.47	1.12–1.94	0.008
Dementia	1.38	1.14–1.67	<0.001
Charlson Comorbidity Index	1.12	1.10–1.15	<0.001
Age	1.07	1.06–1.09	<0.001
Hemoglobin upon admission (g/dL)	0.90	0.85–0.93	<0.001
Albumin upon admission (g/dL)	0.65	0.55–0.76	<0.001

Legend: ASA: American Society of Anesthesiology; 95%CI: 95%, Confidence Interval, units missing.

**Table 4:** Multivariate Cox regression analysis with variables during hospital admission and at discharge.

Variable	Hazard Ratio	95%CI	*p*-Value
Respiratory complication	2.20	1.66–2.80	<0.001
Hospital readmission	2.17	1.86–2.52	<0.001
Cardiac complication	1.64	1.28–2.10	<0.001
Delirium during admission	1.38	1.12–1.69	0.002
Complications during admission	1.31	1.12–1.54	0.001
Glomerular filtration rate at discharge (mL/min)	995	0.993–0.998	<0.001
Albumin at discharge (mg/dL)	0.59	0.51–0.67	<0.001

Legend: 95%CI: 95% Confidence Interval, units missing.

**Table 5:** Multivariate Cox regression analysis with all variables at admission and discharge.

Variable	Hazard Ratio	95%CI	*p*-Value
Respiratory complications	2.42	1.42–4.14	0.001
Hospital readmission	1.96	1.66–2.32	<0.001
Cardiac complication	1.88	1.46–2.42	<0.001
Male sex	1.65	1.40–1.96	<0.001
Dementia	1.37	1.13–1.66	<0.001
ASA score ≥3	1.34	1.02–1.77	0.036
Charlson Comorbidity Index	1.08	1.05–1.12	<0.001
Age	1.08	1.06–1.09	<0.001
Hemoglobin upon admission (g/dL)	0.89	0.85–0.93	<0.001
Albumin at discharge (mg/dL)	0.64	0.56–0.74	<0.001

Legend: 95%CI: 95% Confidence Interval; ASA: American Society of Anesthesiology, units are missing.

## Data Availability

The data presented in this study are available on request from the principal investigator.
